# Prognostic value of the triglyceride-glucose index for major adverse events in acute aortic dissection patients

**DOI:** 10.3389/fcvm.2025.1737368

**Published:** 2026-01-09

**Authors:** Bo Zhang, Yanda Zhang, Changwei Zhou, Rongyi Zheng, Yanwei Zhang, Long Wang

**Affiliations:** Department of Cardiothoracic Surgery, Henan Chest Hospital, Affiliated Hospital of Zhengzhou University, Zhengzhou, China,

**Keywords:** aortic dissection, insulin resistance, major adverse cardiac and cerebrovascular events, prognosis, risk, triglyceride-glucose index

## Abstract

**Background:**

The prognostic value of insulin resistance (IR) indices in patients with acute aortic dissection (AAD) remains unclear. This study investigated the associations between four IR indices and major adverse cardiac and cerebrovascular events (MACCEs) in AAD patients.

**Methods:**

We retrospectively analyzed 114 AAD patients from Henan Chest Hospital (December 2017-December 2023). Four IR indices were calculated: triglyceride-glucose (TyG) index, TyG-BMI index, TG/HDL-C ratio, and METS-IR. Multivariable logistic regression models were constructed to evaluate associations with MACCEs. Subgroup analyses were performed in male, hypertensive, diabetic, and smoking patients.

**Results:**

Among 114 patients (mean age 50.67 ± 12.49 years), 55 (48.2%) developed MACCEs. In fully adjusted models, only TyG index independently predicted MACCEs (OR = 2.46, 95% CI: 1.24–4.90, *p* = 0.010). Subgroup analyses revealed robust associations in male patients (OR = 2.87, 95% CI: 1.24–6.65, *p* = 0.014) and smokers (OR = 3.13, 95% CI: 1.05–9.31, *p* = 0.040), but not in hypertensive patients.

**Conclusions:**

An elevated TyG index independently predicts MACCEs in AAD patients, particularly in males and smokers, and may support risk stratification and intensified follow-up in high-risk individuals.

## 1 Introduction

Acute aortic dissection (AAD) represents one of the most catastrophic cardiovascular emergencies, characterized by a tear in the intimal layer of the aorta that allows blood to dissect through the layers of the aortic wall (1). Despite advances in diagnostic imaging and surgical techniques, AAD remains associated with devastating outcomes, with mortality rates increasing by 1%–2% per hour if left untreated (2). The in-hospital mortality rate for surgically managed type A dissections ranges from 15% to 30%, while long-term survival remains suboptimal, with 5-year survival rates of approximately 70%–80% and 10-year survival rates declining to 50%–60% (3). These sobering statistics underscore the critical need for improved risk stratification tools to identify patients at higher risk of adverse outcomes. Several risk factors for AAD have been well established, including advanced age, male gender, hypertension, atherosclerosis, and connective tissue disorders such as Marfan syndrome (4, 5). Recent epidemiological studies have identified additional metabolic risk factors, with smoking habit, elevated systolic and diastolic blood pressure, higher non-HDL cholesterol, and lower HDL cholesterol levels demonstrating strong associations with mortality from aortic diseases. However, the relationship between metabolic dysfunction and AAD outcomes remains incompletely understood. Interestingly, while traditional cardiovascular risk factors predict AAD occurrence, their role in determining post-dissection prognosis is less well characterized, particularly for metabolic parameters.

Insulin resistance (IR), a fundamental feature of metabolic syndrome and type 2 diabetes mellitus, has emerged as a critical regulator in the development and progression of cardiovascular diseases (6, 7). IR is intrinsically linked to multiple cardiovascular risk factors, including hypertension, dyslipidemia, and hyperglycemia, collectively contributing to endothelial dysfunction, oxidative stress, and chronic inflammation (8). Recent experimental evidence suggests that IR may promote aortic dissection formation through phenotypic switching of vascular smooth muscle cells, with the majority of patients with acute thoracic aortic dissection exhibiting elevated markers of IR (9). However, the prognostic significance of IR in patients who have already developed AAD remains poorly defined, representing a significant gap in current knowledge. Traditional assessment of IR, such as the hyperinsulinemic-euglycemic clamp technique or homeostasis model assessment (HOMA-IR), requires insulin measurements that are often unavailable in emergency settings or resource-limited environments (10). Consequently, surrogate markers of IR that can be calculated from routine laboratory tests have gained considerable attention. Among these, the triglyceride-glucose (TyG) index has demonstrated superior performance as a simple, cost-effective, and reliable biomarker of IR (11). Accumulating evidence has established strong associations between elevated TyG index and adverse cardiovascular outcomes across diverse populations and disease states, including coronary artery disease, heart failure, and stroke (11–13).

Recent meta-analyses have confirmed that elevated TyG index independently predicts major adverse cardiovascular events (MACEs) in patients with coronary artery disease, with individuals in higher TyG index quartiles exhibiting a 2.14-fold increased risk compared to those with lower values (14). Beyond the TyG index, other IR surrogates have been proposed, including the TyG-BMI index (TyG index multiplied by body mass index), the TG/HDL-C ratio, and the Metabolic Score for Insulin Resistance (METS-IR) (15, 16). These indices incorporate anthropometric and lipid parameters to provide enhanced assessment of metabolic dysfunction. However, their comparative prognostic value in predicting outcomes following AAD has not been systematically evaluated. Despite the established role of IR surrogates in predicting cardiovascular events in various clinical contexts, their utility in risk stratifying patients with AAD remains unexplored. Given the high mortality associated with AAD and the routine availability of triglycerides and glucose measurements, investigating whether simple IR indices can identify patients at increased risk of major adverse cardiac and cerebrovascular events (MACCEs) following AAD would have important clinical implications. Such tools could facilitate early identification of high-risk individuals requiring intensive monitoring and aggressive therapeutic interventions.

Therefore, this study aimed to investigate the associations between four IR indices (TyG index, TyG-BMI index, TG/HDL-C ratio, and METS-IR) and MACCEs in patients with AAD. Additionally, we performed subgroup analyses in clinically relevant populations, including male patients and those with hypertension, diabetes mellitus, or smoking history, to identify patient subgroups in whom IR indices might have enhanced prognostic value. We hypothesized that elevated IR indices, particularly the TyG index, would be independently associated with increased risk of MACCEs following AAD.

## 2 Methods

### 2.1 Study design and population

This retrospective cohort study was conducted at Henan Chest Hospital between December 2017 and December 2023. During this period, 233 consecutive patients with suspected acute aortic dissection (AAD) were initially screened. After diagnostic imaging, 51 patients were excluded due to the absence of AAD confirmation by computed tomography angiography (CTA). Among the remaining patients with confirmed AAD, 10 were further excluded because of chronic aortic dissection or a history of prior aortic surgery, and 28 were excluded owing to incomplete clinical or imaging data or loss to follow-up. Ultimately, 144 patients with confirmed acute aortic dissection were included in the final analysis. The diagnosis of AAD was established using CTA in accordance with established diagnostic criteria.

All included patients were followed for the occurrence of major adverse cardiac and cerebrovascular events (MACCEs) during hospitalization and after discharge. The study protocol was approved by the Ethics Committee of Henan Chest Hospital, and the requirement for written informed consent was waived due to the retrospective nature of the study.

### 2.2 Data collection

Baseline demographic and clinical data were extracted from electronic medical records, including age, sex, body mass index (BMI), and medical history of hypertension, hyperlipidemia, diabetes mellitus, coronary artery disease (CAD), prior stroke, smoking status, and alcohol consumption. Laboratory parameters were obtained within 24 h of hospital admission, including fasting blood glucose, triglycerides (TG), high-density lipoprotein cholesterol (HDL-C), low-density lipoprotein cholesterol (LDL-C), white blood cell count (WBC), D-dimer, cardiac troponin, creatine kinase-MB (CK-MB), albumin, alanine aminotransferase (ALT), aspartate aminotransferase (AST), and creatinine. Left ventricular ejection fraction (EF) was assessed by transthoracic echocardiography. The primary outcome was the occurrence of MACCEs during the follow-up period. Patients were categorized into two groups: the MACCEs group and the no-MACCEs group.

### 2.3 Univariate and multivariate analysis

Baseline characteristics, medical history, and laboratory parameters were compared between the MACCEs and no-MACCEs groups using appropriate statistical tests. Variables showing significant differences in univariate analyses were subsequently entered into multivariate logistic regression models to identify independent predictors of MACCEs. A forest plot was constructed to visualize the odds ratios (ORs) and 95% confidence intervals (CIs) of significant predictors from the multivariate analysis.

### 2.4 Definition of insulin resistance indices

Based on previous studies, four surrogate markers of insulin resistance were calculated: (1) Triglyceride-glucose (TyG) index = ln [TG (mg/dL) × fasting glucose (mg/dL)/2]; (2) TyG-BMI index = TyG index × BMI; (3) TG/HDL-C ratio = TG (mmol/L)/HDL-C (mmol/L); and (4) Metabolic Score for Insulin Resistance (METS-IR) = ln [(2 × fasting glucose + TG) × BMI]/[ln (HDL-C)].

### 2.5 Association between insulin resistance indices and MACCEs

To evaluate the association between insulin resistance indices and MACCEs, we constructed three sequential logistic regression models: Model 1 (unadjusted); Model 2 (adjusted for sex, age, hypertension, hyperlipidemia, diabetes mellitus, stroke history, smoking, and alcohol consumption); and Model 3 (further adjusted for EF, LDL-C, WBC, D-dimer, troponin, CK-MB, albumin, ALT, AST, and creatinine). Results were presented as ORs with 95% CIs.

### 2.6 Subgroup analyses

Stratified analyses were performed in clinically relevant subgroups, including male patients, patients with hypertension, patients with diabetes mellitus, and smokers. The associations between insulin resistance indices and MACCEs were assessed within each subgroup using the same three-model adjustment strategy.

### 2.7 Receiver operating characteristic curve analysis

ROC curves were constructed to assess the discriminative ability of insulin resistance indices for predicting MACCEs in the overall cohort and in each subgroup (male patients, hypertension patients, diabetes mellitus patients, and smoking patients). A basic model containing traditional cardiovascular risk factors was established, and the incremental predictive value of adding each insulin resistance index was evaluated. The area under the curve (AUC) was calculated to quantify predictive performance.

To derive a clinically applicable threshold, the optimal cut-off value of the TyG index was determined using the Youden index, defined as the maximum value of sensitivity plus specificity minus one. The TyG index value corresponding to the highest Youden index was considered the optimal cut-off for MACCE risk stratification.

### 2.8 Statistical analysis

Continuous variables were expressed as mean ± standard deviation for normally distributed data. Categorical variables were presented as frequencies and percentages. Differences between groups were compared using Student's *t*-test, Mann–Whitney *U*-test, chi-square test, or Fisher's exact test, as appropriate. All statistical analyses were performed using R software. A two-sided *p*-value < 0.05 was considered statistically significant.

## 3 Results

### 3.1 Patient characteristics and group comparisons

**Figure 1:**
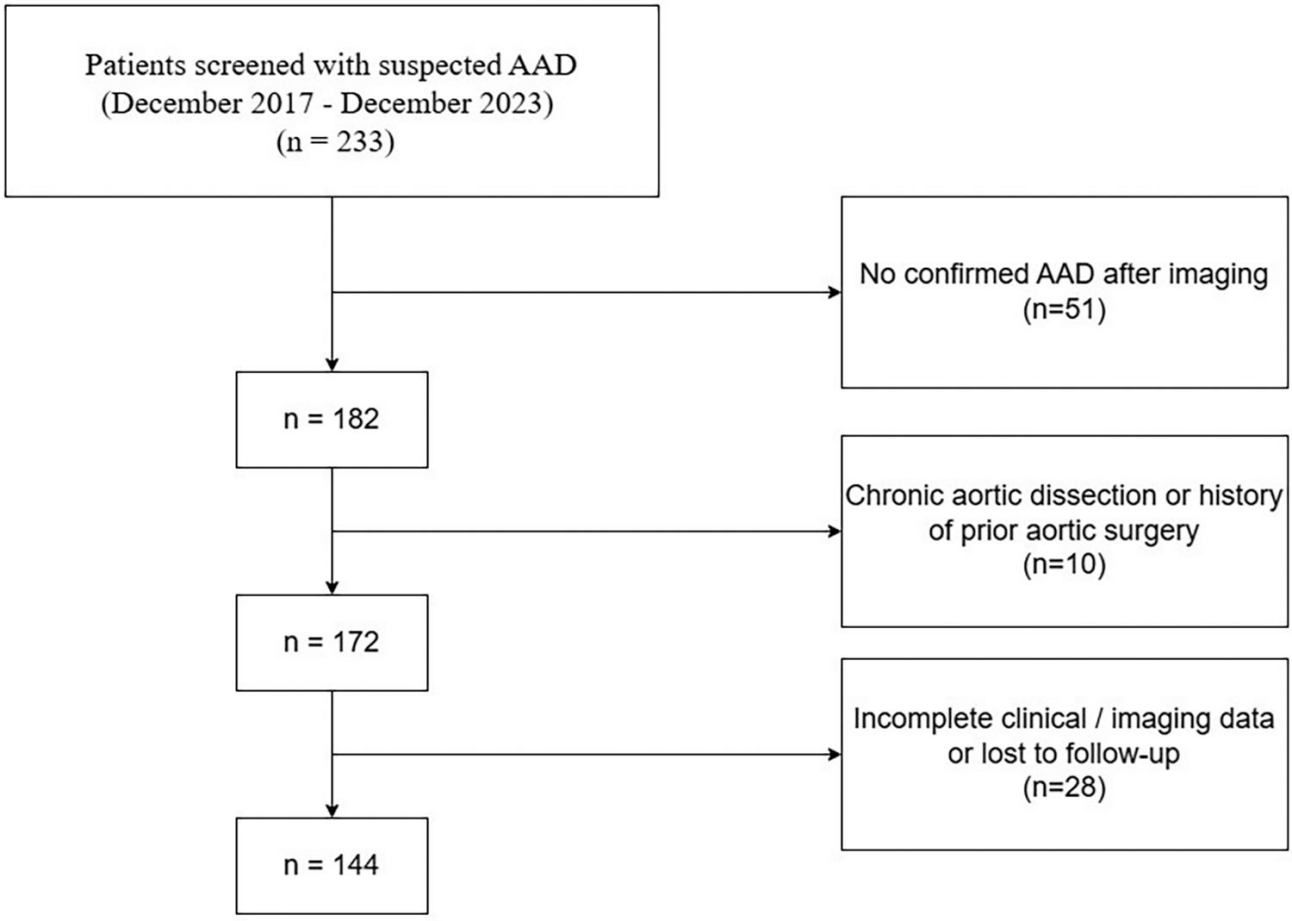
The flowchart of the research.

The overall workflow of the study is illustrated in Figure 1. A total of 114 patients with acute aortic dissection were included in this study, with a mean age of 50.67 ± 12.49 years. Among them, 55 patients (48.2%) developed MACCEs during the follow-up period, including 45 events were aortic rupture-related multiple organ ischemia, 4 were postoperative stroke, 5 were intraoperative myocardial infarction, and 1 was postoperative reintervention. Median follow-up duration was 13.1 months. Table 1 presents the baseline characteristics of patients stratified by MACCEs occurrence. Patients in the MACCEs group were more likely to be male (85.5% vs. 66.1%, *p* = 0.029) and had higher BMI (27.31 ± 2.62 vs. 26.11 ± 2.95 kg/m^2^, *p* = 0.024) compared to the no-MACCEs group. Regarding medical history, the prevalence of diabetes mellitus was significantly higher in the MACCEs group (67.3% vs. 37.3%, *p* = 0.003), while no significant differences were observed in hypertension, hyperlipidemia, CAD, stroke history, smoking, or alcohol consumption between the two groups. Laboratory examination revealed that patients with MACCEs had elevated levels of glycemia (6.60 ± 2.00 vs. 5.85 ± 1.50 mmol/L, *p* = 0.026), triglycerides (1.76 ± 0.91 vs. 1.38 ± 1.02 mmol/L, *p* = 0.037), WBC (16.12 ± 7.25 vs. 12.13 ± 4.55 mmol/L, *p* = 0.001), D-dimer (18.60 ± 17.40 vs. 11.28 ± 11.77 mmol/L, *p* = 0.009), ALT (156.63 ± 358.05 vs. 54.70 ± 63.65 U/L, *p* = 0.034), AST (209.71 ± 465.40 vs. 68.45 ± 111.05 U/L, *p* = 0.025), and creatinine (163.11 ± 138.07 vs. 93.19 ± 41.38 µmol/L, *p* < 0.001). No significant differences were found in HDL-C, LDL-C, troponin, CK-MB, albumin, or ejection fraction between the two groups.

**Table 1:** Baseline clinical characteristics of patients.

Characteristics	no-MACCEs	MACCEs	Total	*P* value
	(*n* = 59)	(*n* = 55)	(*n* = 114)	
Baseline characteristics				
Age (years)	49.46 ± 11.98	51.96 ± 13.00	50.67 ± 12.49	0.286
Male Sex, *n* (%)	39 (66.1)	47 (85.5)	95 (83.3)	0.029
BMI (kg/m^2^)	26.11 ± 2.95	27.31 ± 2.62	26.69 ± 2.85	0.024
Medical history				
Hypertension, *n* (%)	37 (62.7)	44 (80.0)	81 (71.1)	0.068
Hyperlipemia, *n* (%)	19 (32.2)	23 (41.8)	42 (36.8)	0.385
Diabetes mellitus, *n* (%)	22 (37.3)	37 (67.3)	59 (51.8)	0.003
CAD, *n* (%)	29 (49.2)	32 (58.2)	61 (53.5)	0.437
Stroke history	5 (8.5)	5 (9.1)	10 (8.8)	1.000
Smoking, *n* (%)	45 (76.3)	32 (58.2)	77 (67.5)	0.063
Alcohol, *n* (%)	28 (47.5)	24 (43.6)	52 (45.6)	0.825
Laboratory data				
Glycemia, mmol/L	5.85 ± 1.50	6.60 ± 2.00	6.21 ± 1.79	0.026
TG, mmol/L	1.38 ± 1.02	1.76 ± 0.91	1.56 ± 0.98	0.037
HDL-C, mmol/L	1.23 ± 0.41	1.23 ± 0.37	1.23 ± 0.39	0.975
LDL-C, mmol/L	2.49 ± 0.80	2.34 ± 0.76	2.42 ± 0.78	0.316
WBC, mmol/L	12.13 ± 4.55	16.12 ± 7.25	14.06 ± 6.3	0.001
D_dimer, mmol/L	11.28 ± 11.77	18.60 ± 17.40	14.81 ± 15.15	0.009
Troponin, mmol/L	0.32 ± 1.78	0.13 ± 0.31	0.23 ± 1.29	0.446
CK-MB, mmol/L	6.03 ± 13.36	24.99 ± 82.08	15.18 ± 58.32	0.083
Albumin, g/L	38.06 ± 7.76	39.99 ± 5.96	38.99 ± 6.98	0.140
ALT, U/L	54.70 ± 63.65	156.63 ± 358.05	103.88 ± 256.83	0.034
AST, U/L	68.45 ± 111.05	209.71 ± 465.40	136.6 ± 338.91	0.025
Creatinine, µmol/L	93.19 ± 41.38	163.11 ± 138.07	126.92 ± 105.93	<0.001
EF, (%)	63.29 ± 5.80	63.17 (5.30)	62.89 ± 6.11	0.479

MACCEs, major adverse cardiac and cerebrovascular events; BMI, body mass index; CAD, coronary artery disease; TG, triglyceride; HDL-C, high-density lipoprotein cholesterol; LDL-C, low-density lipoprotein cholesterol; WBC, white blood cell count; CK-MB, creatine kinase-MB; ALT, alanine aminotransferase; AST, aspartate aminotransferase; EF, ejection fraction.

### 3.2 Male sex as an independent predictor of MACCEs

**Figure 2:**
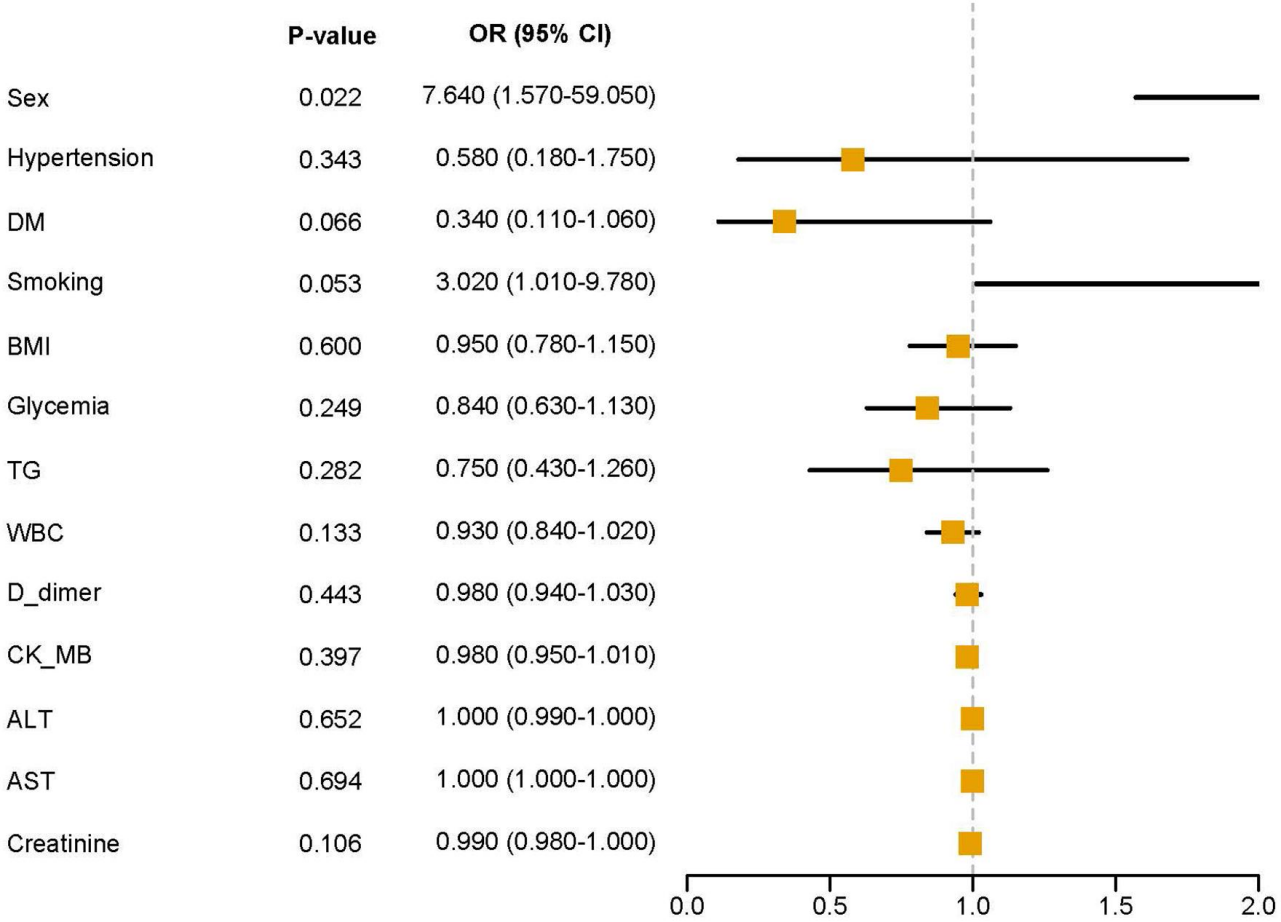
Multivariate analysis forest plot.

Variables showing significant differences in baseline characteristics were entered into multivariate logistic regression analysis. Figure 2 displays the forest plot of multivariate analysis results. After adjusting for potential confounders, male sex remained an independent predictor of MACCEs with an odds ratio of 7.640 (95% CI: 1.570–59.050, *p* = 0.022). Smoking showed a marginally significant association (OR = 3.020, 95% CI: 1.010–9.780, *p* = 0.053).

### 3.3 TyG index independently predicts MACCEs in all patients

Table 2 presents the associations between four insulin resistance indices and MACCEs in all patients across three sequential adjustment models. In the unadjusted model (Model 1), TyG index (OR = 2.18, 95% CI: 1.39–3.42, *p* = 0.001), TyG-BMI index (OR = 1.99, 95% CI: 1.30–3.05, *p* = 0.002), and METS-IR (OR = 1.65, 95% CI: 1.10–2.47, *p* = 0.015) were significantly associated with increased risk of MACCEs, while TG/HDL-C ratio showed no significant association. After adjusting for demographic and clinical variables in Model 2, TyG index (OR = 2.17, 95% CI: 1.28–3.67, *p* = 0.004) and TyG-BMI index (OR = 1.89, 95% CI: 1.12–3.21, *p* = 0.017) remained significantly associated with MACCEs. In the fully adjusted Model 3, only TyG index maintained a significant association with MACCEs (OR = 2.46, 95% CI: 1.24–4.90, *p* = 0.010).

**Table 2:** Clinical features identified by univariate analysis.

Characteristics	OR (95% CI) *1	*P* value	OR (95% CI) *2	*P* value	OR (95% CI) *3	*P* value
TyG index	2.18 (1.39–3.42)	0.001	2.17 (1.28–3.67)	0.004	2.46 (1.24–4.90)	0.010
TyG-BMI index	1.99 (1.30–3.05)	0.002	1.89 (1.12–3.21)	0.017	1.75 (0.91–3.37)	0.094
TG/HDL-C ratio	1.34 (0.89–2.03)	0.162	1.26 (0.80–1.98)	0.322	1.59 (0.91–2.78)	0.101
METS-IR	1.65 (1.10–2.47)	0.015	1.41 (0.88–2.28)	0.156	1.37 (0.76–2.48)	0.293

Model 1: unadjusted.

Model 2: adjusted for Sex, Age, Hypertension, Hyperlipemia, Diabetes mellitus, Stroke history, Smoking, and Alcohol.

Model 3: adjusted for EF, LDL-C, WBC, D-dimer, Troponin, CK-MB, Albumin, ALT, AST, and Creatinine.

To further enhance clinical interpretability, receiver operating characteristic analysis with Youden index optimization was performed to determine an optimal TyG index threshold. A TyG value of 8.64 was identified as the optimal cut-off, yielding a sensitivity of 78.2% and a specificity of 61.0% for predicting MACCEs. When patients were stratified according to this cut-off, those in the high-TyG group (TyG ≥ 8.64) exhibited a significantly higher risk of MACCEs compared with those in the low-TyG group.

### 3.4 TyG index shows robust association in male and smoking patients

Subgroup analyses were performed to explore the associations in clinically relevant populations. As shown in Supplementary Table S1, in male patients, TyG index demonstrated the strongest and most consistent association with MACCEs across all three models, with ORs of 3.27 (95% CI: 1.77–6.06, *p* < 0.001), 3.43 (95% CI: 1.68–7.02, *p* = 0.001), and 2.87 (95% CI: 1.24–6.65, *p* = 0.014) in Models 1, 2, and 3, respectively. TyG-BMI index also showed significant associations in Models 1 and 2. Supplementary Table S3 demonstrates that in patients with diabetes mellitus, TyG index was significantly associated with MACCEs in Model 1 (OR = 2.00, 95% CI: 1.05–3.80, *p* = 0.035) and Model 2 (OR = 2.25, 95% CI: 1.08–4.70, *p* = 0.030), but the association became non-significant after full adjustment in Model 3. As presented in Supplementary Table S4, among smoking patients, TyG index showed robust associations with MACCEs in all three models, with ORs of 2.84 (95% CI: 1.52–5.28, *p* = 0.001), 2.81 (95% CI: 1.30–6.07, *p* = 0.009), and 3.13 (95% CI: 1.05–9.31, *p* = 0.040). Interestingly, Supplementary Table S2 shows that in hypertensive patients, none of the insulin resistance indices showed significant associations with MACCEs after multivariate adjustment.

### 3.5 Insulin resistance indices demonstrate good discriminative ability for MACCEs

**Figure 3:**
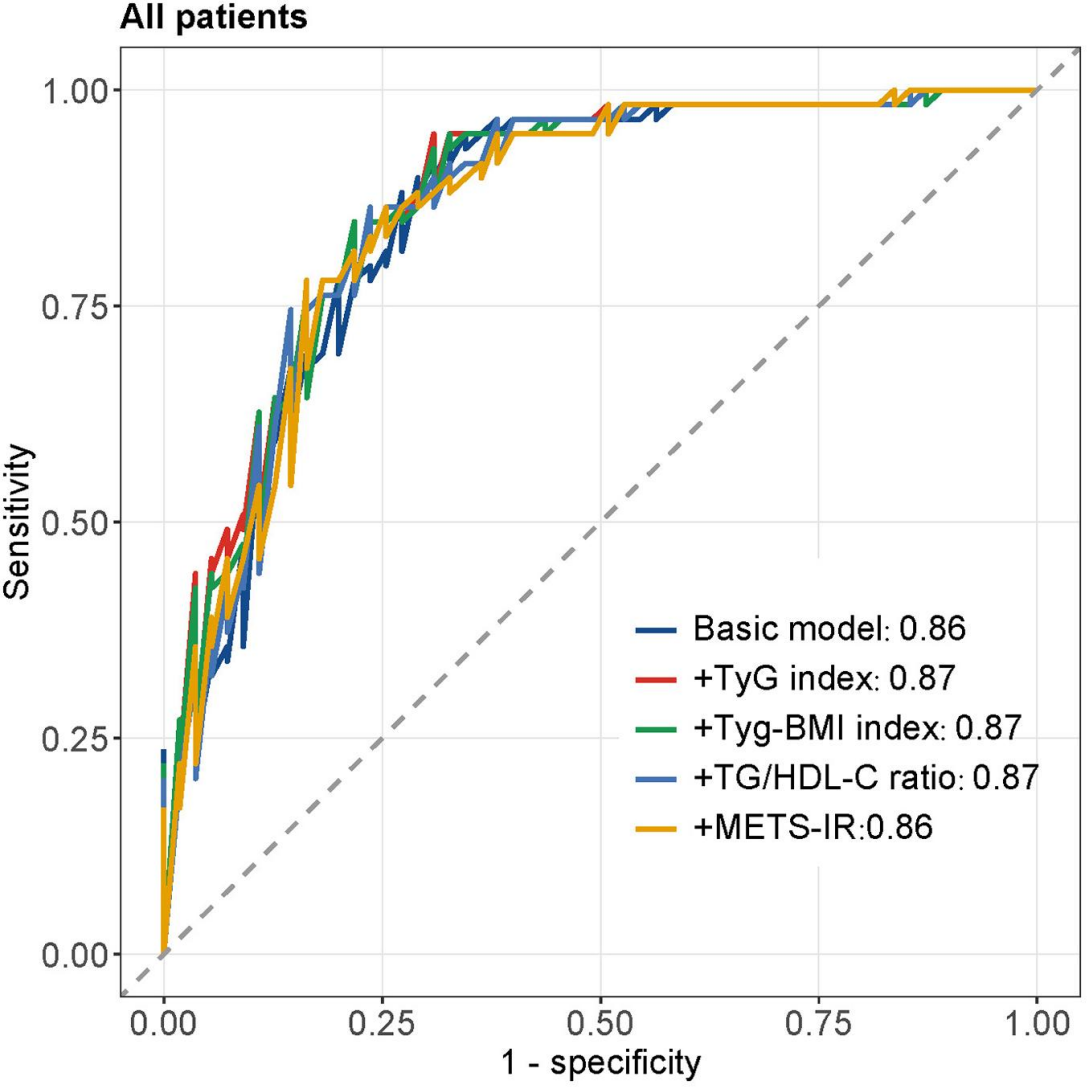
The ROC curves of all patients.

**Figure 4:**
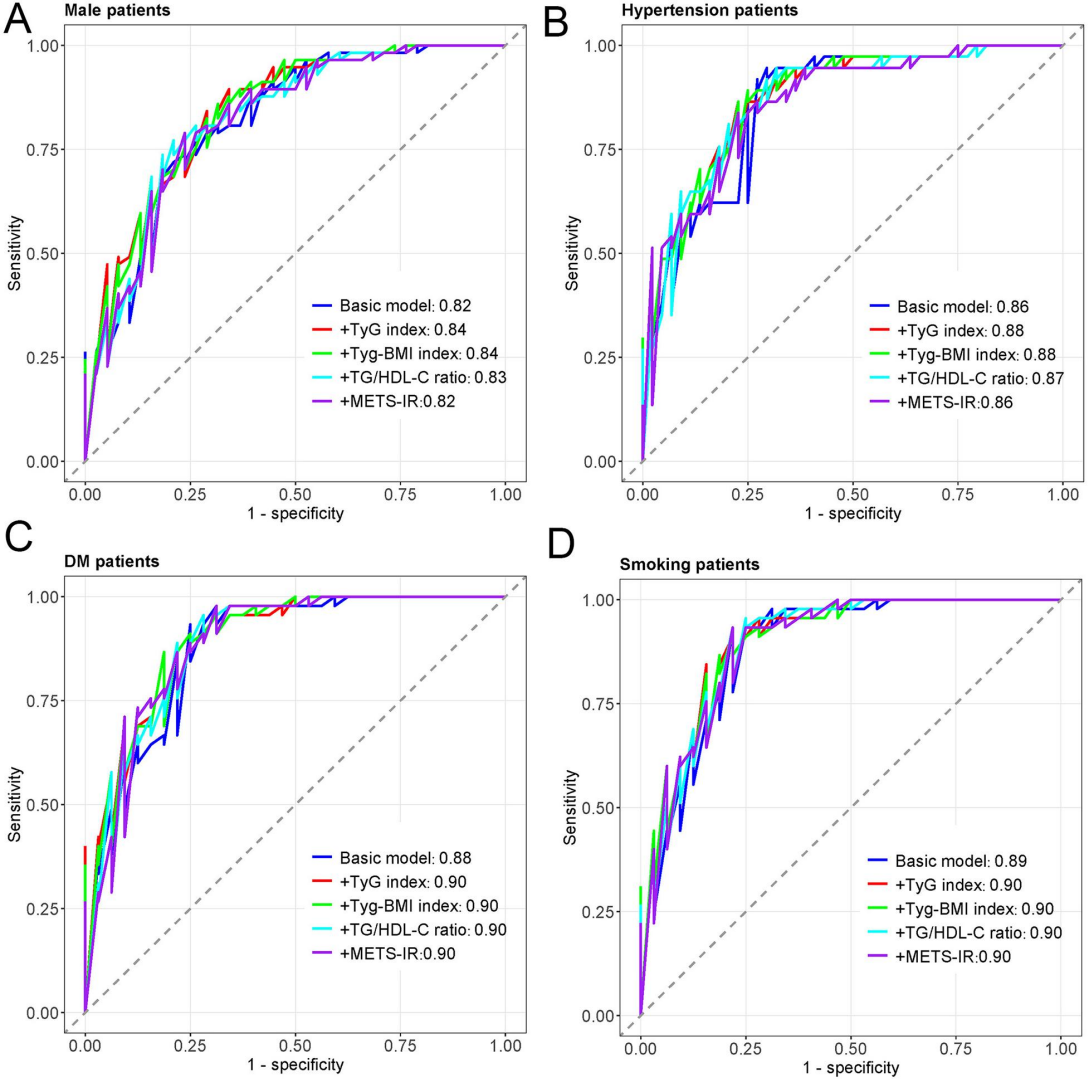
The ROC curves of subtype patients. **(A)** The ROC curves of male patients. **(B)** The ROC curves of Hypertension patients. **(C)** The ROC curves of DM patients. **(D)** The ROC curves of smoking patients.

Figure 3 displays the ROC curves for different insulin resistance indices in predicting MACCEs in all patients. The basic model (AUC = 0.86) incorporating traditional cardiovascular risk factors demonstrated good discriminative ability. Adding TyG index (AUC = 0.87), TyG-BMI index (AUC = 0.87), TG/HDL-C ratio (AUC = 0.87), or METS-IR (AUC = 0.86) to the basic model showed comparable predictive performance, with the curves closely overlapping. Figure 4 presents subgroup ROC analyses, revealing similar patterns in male patients (Figure 4A), hypertension patients (Figure 4B), diabetes mellitus patients (Figure 4C), and smoking patients (Figure 4D), with all indices showing acceptable discriminative ability for predicting MACCEs in these specific populations.

## 4 Discussion

This study provides novel evidence that elevated insulin resistance indices, particularly the TyG index, independently predict MACCEs in patients with acute aortic dissection. To our knowledge, this represents the first comprehensive investigation evaluating multiple IR surrogates for prognostic stratification in AAD patients. Our principal findings demonstrate that the TyG index maintained a significant association with MACCEs even after adjustment for traditional cardiovascular risk factors and laboratory parameters, with ORs ranging from 2.18 to 2.46 across different models. Importantly, subgroup analyses revealed that this association was particularly robust in male patients and current smokers, suggesting potential population-specific applications for risk stratification. These findings align with extensive evidence demonstrating the prognostic utility of the TyG index across diverse cardiovascular conditions. Previous meta-analyses have consistently shown that elevated TyG index predicts adverse outcomes in patients with coronary artery disease, with individuals in higher TyG quartiles exhibiting more than twofold increased risk of major adverse cardiovascular events (14, 17). The magnitude of association observed in our AAD cohort is comparable to that reported for established biomarkers such as D-dimer and inflammatory markers, suggesting that the TyG index could serve as a complementary risk stratification tool in clinical practice (18).

Several interconnected pathophysiological mechanisms may explain the association between IR and adverse outcomes in AAD patients. First, IR induces endothelial dysfunction through multiple pathways, including reduced nitric oxide bioavailability, enhanced oxidative stress, and increased production of reactive oxygen species (8, 19). These processes compromise vascular integrity and promote inflammatory cascades that may exacerbate aortic wall injury following dissection. Studies have demonstrated that IR-related endothelial dysfunction disrupts the balance between the PI3K-NO vasodilatory pathway and the MAPK-endothelin-1 vasoconstrictive pathway, leading to impaired vascular homeostasis (20). Second, IR is intimately linked with systemic inflammation, as evidenced by elevated levels of pro-inflammatory cytokines such as interleukin-6 and tumor necrosis factor-alpha (21). Recent investigations have shown that inflammation partially mediates the relationship between IR and cardiovascular events, suggesting a synergistic detrimental effect (22). In the context of AAD, heightened inflammatory responses may accelerate complications such as organ malperfusion, cardiac tamponade, and thrombotic events. Third, IR promotes activation of the renin-angiotensin-aldosterone system and sympathetic nervous system, resulting in persistent hemodynamic stress on the already compromised aortic wall (23). This increased hemodynamic burden, coupled with metabolic dysfunction, may predispose patients to extension of dissection, false lumen expansion, or even aortic rupture. The convergence of these mechanisms creates a pathophysiological milieu that substantially increases the risk of adverse cardiovascular outcomes in AAD patients with elevated insulin resistance.

The differential associations observed across subgroups provide important insights into risk stratification. The particularly strong association between TyG index and MACCEs in male patients deserves careful consideration. These findings are consistent with established sex differences in insulin resistance and cardiovascular risk. Although females generally maintain better insulin sensitivity during reproductive years, the relationship between IR and cardiovascular events appears attenuated in women compared to men (24, 25). This “female advantage” has been attributed to protective effects of estrogen, which enhances insulin signaling, reduces oxidative stress, and improves endothelial function (26). Conversely, males demonstrate stronger associations between metabolic dysfunction and cardiovascular outcomes, potentially due to greater visceral adiposity, higher inflammatory burden, and absence of estrogen's cardioprotective effects (27). Similarly, the robust association observed in smoking patients (ORs exceeding 3.0) is biologically plausible, as tobacco exposure induces insulin resistance through increased oxidative stress, chronic inflammation, and altered adipokine secretion, synergizing with IR to accelerate thrombotic complications (28). Interestingly, none of the IR indices showed significant associations with MACCEs in hypertensive patients after multivariate adjustment. This paradoxical finding may reflect the complex interplay between hypertension, IR, and aortic pathology, where the overwhelming hemodynamic burden imposed by elevated blood pressure may overshadow the additional risk conferred by metabolic dysfunction (29). Alternatively, intensive blood pressure management in hypertensive AAD patients may have effectively mitigated IR-related risks. Finally, the coexistence of hypertension and insulin resistance may introduce collinearity and risk saturation effects, limiting the ability of IR indices to further discriminate prognosis within this subgroup. These subgroup-specific findings emphasize that male AAD patients and smokers with elevated TyG index represent particularly high-risk populations warranting intensive surveillance and aggressive risk factor modification.

From a clinical perspective, the identification of a Youden index–derived TyG threshold enables translation of our findings into practical management strategies. Patients with elevated TyG index (≥8.64), particularly males and current smokers, may benefit from intensified metabolic surveillance, including more frequent monitoring of fasting glucose and triglycerides during follow-up, alongside optimization of lipid-lowering and glucose-lowering therapies according to high-risk cardiovascular targets. In these high-risk subgroups, closer imaging surveillance of the aorta and structured lifestyle interventions-especially smoking cessation-may be warranted. Importantly, the fully adjusted effect size observed for the TyG index (OR = 2.46) is comparable to that reported in meta-analyses of TyG index in other cardiovascular diseases, such as acute coronary syndrome and ischemic stroke, and is of similar magnitude to established biomarkers used in acute aortic syndromes, including D-dimer. These findings suggest that the TyG index provides clinically meaningful and complementary prognostic information rather than a marginal incremental signal.

This study highlights the TyG index as a simple, readily available, and cost-effective prognostic marker for patients with acute aortic dissection. Unlike insulin-based indices, the TyG index relies solely on routine fasting glucose and triglyceride measurements, enabling rapid risk stratification without additional testing burden, particularly in acute or resource-limited settings. Importantly, its prognostic value remained consistent across multiple multivariable models, underscoring its robustness. The key innovation of this study lies in demonstrating, for the first time, the independent and stable prognostic utility of the TyG index in AAD, supporting its potential integration into existing risk assessment frameworks to improve early identification of high-risk patients and guide individualized management strategies. Several limitations should be acknowledged. First, the retrospective single-center design may limit generalizability, and multicenter prospective studies are warranted. Second, insulin resistance indices were assessed only at baseline, and dynamic changes over time were not evaluated. Third, our relatively modest sample size of 114 patients precluded detailed analyses of specific MACCE components individually and may have limited statistical power for subgroup analyses. Future work will focus on increasing the sample size to enhance the robustness of our findings. In addition, information on medication adherence and lifestyle factors during follow-up was unavailable.

In conclusion, an elevated TyG index independently predicts MACCEs in patients with acute aortic dissection, with particularly pronounced associations in male patients and current smokers. Given its derivation from routinely available laboratory parameters, the TyG index provides a practical tool for early risk stratification and may help identify AAD survivors who warrant intensified metabolic monitoring, closer imaging surveillance, and aggressive modification of cardiometabolic risk factors. Future research should prioritize prospective multicenter validation, evaluation of TyG-guided management strategies, and investigation of whether interventions targeting insulin resistance can translate into improved long-term outcomes in high-risk AAD populations.

## Data Availability

The original contributions presented in the study are included in the article/Supplementary Material, further inquiries can be directed to the corresponding author.
