# Supplementary material for: Prognostic value of the triglyceride-glucose index for major adverse events in acute aortic dissection patients
Source: Front Cardiovasc Med. 2026 Jan 9;12:1737368. doi: 10.3389/fcvm.2025.1737368 (PMC12827543; doi:10.3389/fcvm.2025.1737368)
Supplement: Supplementary file 1 [file Datasheet1.docx]

**Supplementary Tables**

**Table S1** Association between IR indexes and MACCEs in male patients.

|  | OR (95% CI) *1 | P value | OR (95% CI) *2 | P value | OR (95% CI) *3 | P value |
| --- | --- | --- | --- | --- | --- | --- |
| TyG index | 3.27 (1.77–6.06) | 0.000 | 3.43 (1.68–7.02) | 0.001 | 2.87 (1.24–6.65) | 0.014 |
| TyG-BMI index | 2.23 (1.34–3.73) | 0.002 | 2.28 (1.22–4.27) | 0.010 | 2.34 (0.94–5.83) | 0.068 |
| TG/HDL-C ratio | 1.50 (0.88–2.55) | 0.138 | 1.41 (0.80–2.49) | 0.237 | 1.78 (0.86–3.71) | 0.123 |
| METS-IR | 1.62 (1.01–2.59) | 0.044 | 1.45 (0.84–2.51) | 0.183 | 1.52 (0.68–3.42) | 0.309 |

**Table S2** Association between IR indexes and MACCEs in hypertension patients.

|  | OR (95% CI) *1 | P value | OR (95% CI) *2 | P value | OR (95% CI) *3 | P value |
| --- | --- | --- | --- | --- | --- | --- |
| TyG index | 0.79 (0.56-1.11) | 0.172 | 0.68 (0.46-0.99) | 0.046 | 0.70 (0.41-1.21) | 0.205 |
| TyG-BMI index | 1.08 (0.78-1.49) | 0.638 | 0.93 (0.65-1.34) | 0.712 | 0.78 (0.46-1.30) | 0.333 |
| TG/HDL-C ratio | 0.52 (0.27-0.98) | 0.042 | 0.51 (0.25-1.01) | 0.054 | 0.35 (0.11-1.12) | 0.077 |
| METS-IR | 1.02 (0.74-1.41) | 0.905 | 0.93 (0.65-1.33) | 0.681 | 0.75 (0.44-1.27) | 0.280 |

**Table S3** Association between IR indexes and MACCEs in DM patients.

|  | OR (95% CI) *1 | P value | OR (95% CI) *2 | P value | OR (95% CI) *3 | P value |
| --- | --- | --- | --- | --- | --- | --- |
| TyG index | 2.00 (1.05–3.80) | 0.035 | 2.25 (1.08–4.70) | 0.030 | 2.06 (0.79–5.35) | 0.139 |
| TyG-BMI index | 1.75 (0.97–3.14) | 0.061 | 1.97 (1.00–3.86) | 0.050 | 1.52 (0.61–3.80) | 0.372 |
| TG/HDL-C ratio | 1.04 (0.61–1.79) | 0.879 | 1.14 (0.61–2.11) | 0.684 | 1.22 (0.49–3.03) | 0.664 |
| METS-IR | 1.32 (0.76–2.30) | 0.326 | 1.33 (0.69–2.56) | 0.396 | 0.97 (0.38–2.47) | 0.950 |

**Table S4** Association between IR indexes and MACCEs in hypertension patients.

|  | OR (95% CI) *1 | P value | OR (95% CI) *2 | P value | OR (95% CI) *3 | P value |
| --- | --- | --- | --- | --- | --- | --- |
| TyG index | 2.84 (1.52–5.28) | 0.001 | 2.81 (1.30–6.07) | 0.009 | 3.13 (1.05–9.31) | 0.040 |
| TyG-BMI index | 2.90 (1.60–5.27) | 0.000 | 2.62 (1.24–5.53) | 0.011 | 2.43 (0.86–6.89) | 0.094 |
| TG/HDL-C ratio | 1.62 (0.95–2.77) | 0.079 | 1.29 (0.72–2.30) | 0.397 | 1.78 (0.75–4.20) | 0.188 |
| METS-IR | 2.43 (1.41–4.18) | 0.001 | 2.08 (1.06–4.08) | 0.033 | 1.79 (0.69–4.69) | 0.233 |
